# A Review of Amelioration of Awareness About Blood Donation Through Various Effective and Practical Strategies

**DOI:** 10.7759/cureus.46892

**Published:** 2023-10-12

**Authors:** Akshay Dorle, Ujwal Gajbe, Brij Raj Singh, Obaid Noman, Pratibha Dawande

**Affiliations:** 1 Pathology, Datta Meghe Medical College, Datta Meghe Institute of Higher Education and Research, Nagpur, IND; 2 Anatomy, Datta Meghe Medical College, Datta Meghe Institute of Higher Education and Research, Nagpur, IND

**Keywords:** transfusion, voluntary blood donation, donating blood, blood donors, blood donation

## Abstract

Blood donations play a crucial role in medical care; however, the global shortage of donors remains and has a serious impact on medical interventions. The challenges involved include the lack of public awareness of the importance of blood donation, the lack of understanding of the process and eligibility criteria for blood donation, and the lack of comprehensive strategies aimed at raising awareness and participation among potential donors, with particular emphasis on the involvement of young people. It is essential to recognize that blood donation delivers significant benefits to donors and recipients, improves overall health, and ultimately saves lives. Various initiatives, such as blood donation camps, dynamic social media campaigns, and strategic networking of medical professionals, have proved effective in promoting blood donation. In particular, in the event of an emergency, the availability of sufficient blood supplies is increasingly essential, underlining the urgent need to establish and maintain a sustainable blood donor network. An in-depth understanding of the motivation and conservation of donors is crucial in this context, as it is known that demographic factors significantly impact the frequency of blood donation. In addition, ethical and legal considerations require careful attention, highlighting the essential role of obtaining informed consent and ensuring the confidentiality of donors throughout the process. As we look ahead to the evolving landscape, it presents a series of formidable challenges. These challenges encompass the critical necessity to broaden and diversify our donor base, thereby extending and varying our sources of financial support for specific initiatives, organizations, or projects. Moreover, we must proactively harness the opportunities presented by emerging technologies and commit ourselves to closing the information gaps within the existing public knowledge sphere. In summary, the review emphasizes the paramount importance of ongoing efforts to strengthen and enrich donors' engagement through customized strategies and educational outreach.

## Introduction and background

Blood donation is a crucial component of healthcare that saves thousands of lives annually [1]. The global shortage of donors is an essential issue, driven primarily by low blood donation rates. It has significant implications for healthcare systems worldwide, as safe and adequate blood supplies are critical to the success of various medical interventions. It can help us preserve life and improve our society's general condition [2]. Transfusion of blood is an essential medical procedure. Various organizations, including healthcare organizations, blood banks, and non-profit organizations dedicated to blood donation and transfusion services, are formulating numerous strategies to tackle the issue, which include initiatives aimed at raising awareness and motivating individuals to contribute to blood donation [3]. However, despite its importance, the general population must still understand and realize the benefits. Of the 171 countries surveyed by the WHO, 62 have a 100% voluntary unpaid system for blood donation. In an unforced and overdue system, donors and recipients are unspecified [4]. The younger generation represents the most promising source for ensuring a secure and sustainable blood supply in the future [5]. While the process is expeditious and commonly practiced in various medical operations, there is a global shortage of blood donors. According to the WHO, a nation can only provide sufficient blood for patients requiring transfusions through a system based on voluntary, unpaid blood donations. The world has a shortage of blood donors [6-8]. According to the American Red Cross, someone in the United States requires blood every two seconds, and transfusions of blood and blood components have saved thousands of lives [1,8]. The current review assesses the most recent strategies implemented to increase awareness and encourage greater participation among blood donors [9]. It also determines whether young people know and have attitudes toward voluntary blood donation. Understanding these strategies will allow us to identify best practices and enhance our efforts to convince more people to donate blood, thus saving more lives. This review article aims to explore utilizing the most recent strategies, thereby enhancing awareness by applying cutting-edge approaches.

## Review

Method

The findings have been reported following the Preferred Reporting Items for Systematic Review and Meta-Analysis (PRISMA) principles and criteria.

Search Sources/Search Strategy

The literature search was conducted through a review of electronic databases like PubMed, Google Scholar, and Scopus using appropriate keywords such as "Blood donation," "Raising awareness," "Latest strategies in blood donation," "blood donation," "blood transfusion," "blood component," "awareness campaign on blood donation," "challenges in blood donation," "future challenges in blood donation," and "transfusion." We obtained the most pertinent research papers and used them in different arrangements using the boolean operators "AND" and "OR" [10].

Inclusion and Exclusion Criteria

We focused on papers written in the English language relevant to the central questions of this review article, and that are narrative reviews such as randomized clinical trials and observational studies. We, however, excluded papers published in languages other than English, irrelevant to the questions, and related to topics.

Number of Articles Included in the Final Review

**Figure 1:**
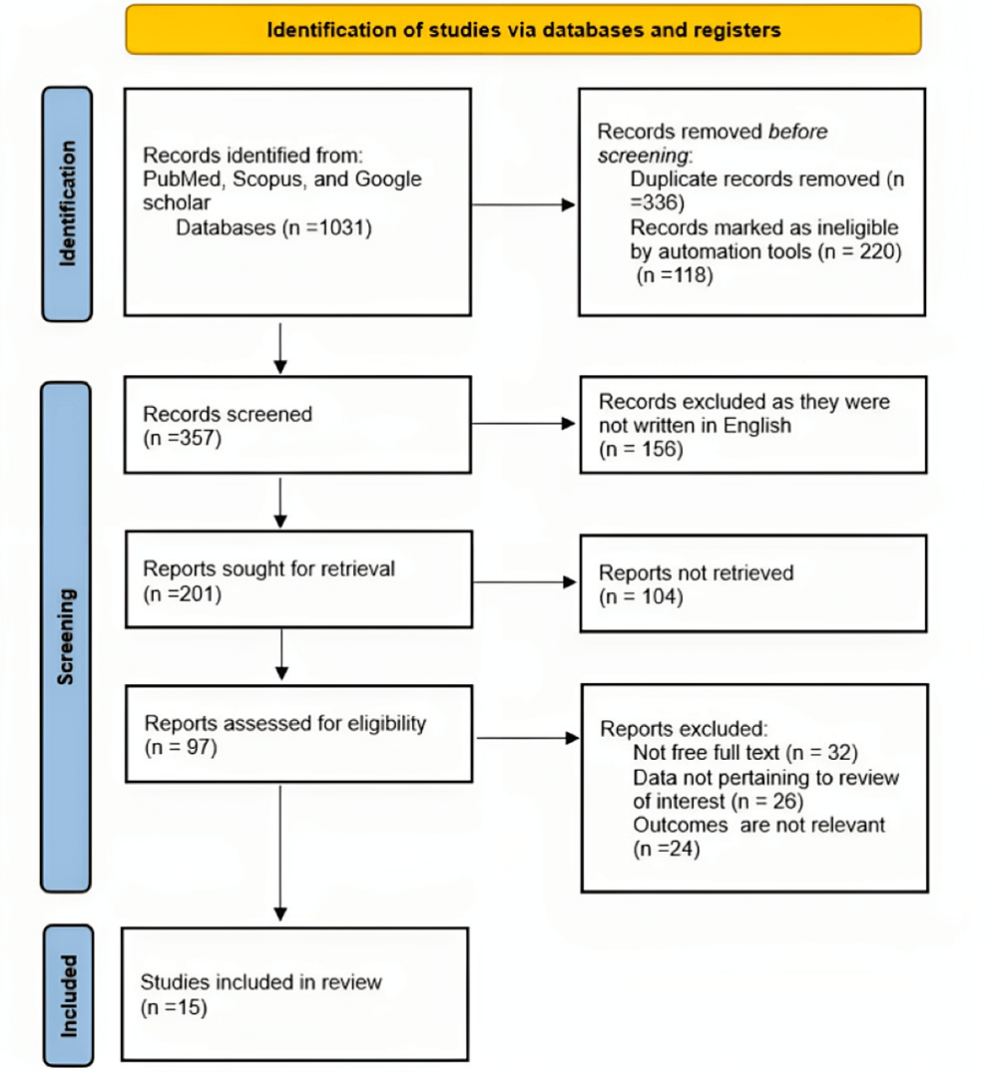
PRISMA chart. n = number of studies; PRISMA: Preferred Reporting Items for Systematic Review and Meta-Analysis.

In the following PRISMA chart, the results show that out of the 1031 records identified, 15 were utilized for studies (Figure 1).

The review included seven articles that discussed using social media and software. Additionally, four articles examined how community-based events can increase blood donation and foster partnerships with various organizations. Furthermore, three articles emphasized regular participation and promotion by actors and players (Table 1).

**Table 1:** Articles included in the study.

Sr No.	Authors	Year	Methodology Employed	Research Findings	Latest Strategies Used
1.	Harrell S et al. [11]	2022	Examined the effect of Facebook's blood donation tool	Blood donation tool increased donations by 4.0% and first-time donors by 18.9%	Social media can increase blood donations and First-time donors are more likely to be influenced by social media
2.	Alanzi T and Alsaeed B [12]	2019	Used WhatsApp to increase blood donation	82% of participants received blood donation requests through social media platforms in Saudi Arabia	Social media can improve blood donation practice
3.	Nabil M et al. [13]	2020	Cloud-Based Medical Monitoring and Web-Based Blood Donation System	The paper discusses the development of a web-based blood donation system that allows donors and patients to offer/request blood donation from blood banks	Enhanced Communication and Coordination Between Blood Donors and Patients through the Integration of Social Media and Software
4.	Phatangare SA et al. [14]	2022	By use of social media sites	Blood donation requests are increasing on social media	By the use of application and social media connects Patients with Nearby Blood Donors
5.	Ramondt S et al. [15]	2021	An Inductive Computational Approach for the Analysis of Social Media Data and Structural Topic Modeling Applied to Facebook and Twitter Data	Twenty-five Topics Grouped into Six Distinct Clusters, Resulting in a Decrease in Messages Announcing Donations, and an Increase in Positive Donation-Related Topics	Blood collection organizations should acknowledge the dynamic nature of social media
6.	Ottong JG et al. [16]	1997	By the hosting community events	Assessment of health institutions facilities for blood transfusion and community education and mobilization for blood donation	Voluntary blood donation can increase blood supply and community involvement improves blood supply situation
7.	Anand N and Pugazhendi I [17]	2018	Cross-sectional study by Multiple logistic regression analysis	45% had good knowledge on blood donation and 33.3% had good practices towards blood donation	By hosting community events, we can significantly enhance blood donation rates
8.	Agrawal A [18]	2016	Social marketing a type of software	Increase in voluntary blood and organ donor	Through the implementation of social marketing strategies and mobile applications, there has been a noticeable increase in voluntary blood and organ donors
9.	Mostafa MA et al. [19]	2014	Development of a Blood Donation System (BDS) a type of software	Convenient blood donation process for donors and medical staff and integration of blood donation centres and health organizations	By harnessing the power of social media and smartphone applications, and leveraging cloud computing and mobile computing technologies, we can enhance the prospects of increasing blood donation rates
10.	McElfresh DC et al. [20]	2020	By use of software	Algorithmic matching of blood donors with donation opportunities and machine learning model trained on prior observations of donor behaviour	Algorithmic matching can increase donor action rate
11.	Zito E et al. [21]	2012	Questionnaire administered to 125 students	Females were found to have greater awareness about blood donation compared to males, and participants were queried about their knowledge of individuals who engage in blood donation	Boosting blood donation rates can be accomplished by involving peer groups and school authorities in recruiting young adult donors, organizing tailored awareness sessions with young staff-donors as presenters, and hosting community events to encourage participation
12.	Alfieri S et al. [21]	2012	Questionnaire administered to students	Two distinct studies were conducted with adolescents. Study I involved 25 participants in five focus groups, facilitating qualitative exploration. Study II administered a self-report questionnaire to 285 adolescents for a comprehensive quantitative perspective, collectively offering insights into adolescent experiences and perspectives on various topics.	Incorporating valuable insights into donor recruitment advertising campaigns, coupled with the hosting of community events, has the potential to drive an increase in blood donation rates
13.	Minguez A and Sese FJ [22]	2022	Emphasis on regular basis	Investigates impact of donation frequency on donation amount has increased blood donation	Higher donation frequencies lead to higher donations
14.	Gemelli CN et al. [23]	2018	Post donation SMS sent to donors who left without making a forward appointment	Receiving SMS messages was associated with higher odds of donors returning to donate within 12 months, demonstrating the effectiveness of SMS in retaining both first-time and experienced donors over the same time frame	Implementing personalized post-donation SMS messages has proven to be an effective strategy for improving donor retention rates, especially among new donors, thus contributing to an increase in blood donations.
15.	Dutta L et al. [24]	2019	Proposed integrated Blood Donation Camp Management System	Blood Donation Camp Management System: designed to manage Blood Donation Campaign effectively	The Blood Donation Camp Management System is specifically designed to enhance the effectiveness of blood donation campaigns, ultimately leading to an increase in blood donations

**Figure 2:**
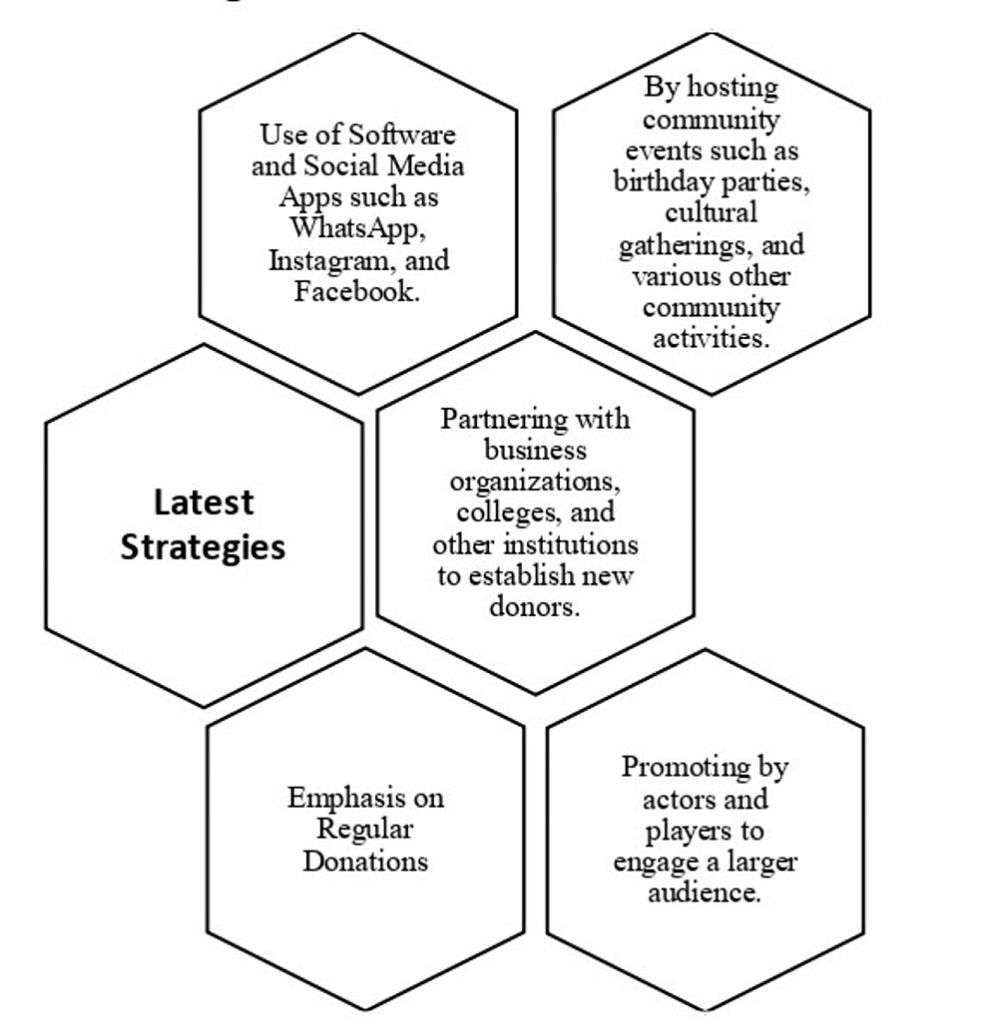
The latest strategies for blood donation.

As per the study referenced, it is evident that the predominant utilization of software and social media applications has led to a notable increase in blood donation rates [11-15,19,20]. Furthermore, successfully implementing blood donation and promotion strategies involves hosting community events, forging partnerships with business organizations, and emphasizing regular engagement [16-18,21]. The involvement of influential public figures, or star personalities, also plays a pivotal role in these efforts [22-24]. This review article provides a comprehensive description of the implications of the latest strategies as detailed in the included articles within Table 2 and Figure 2.

**Table 2:** Implications of latest strategies for blood donation.

The latest strategies for blood donation	Implications of latest strategies for blood donation
Utilization of software and social media applications like WhatsApp, Facebook, and Instagram	The incorporation of software and social media applications, including WhatsApp, Facebook and Instagram has enabled us to establish connections, collaborate, and respond swiftly in times of need. The fusion of digital innovation with the spirit of compassion holds the promise of saving countless lives as we continue to champion the cause of giving the gift of life through the power of software and social media [6].
By hosting community events such as birthday parties, cultural gatherings, and various other community activities	Hosting community events that champion blood donation, whether through birthday parties, cultural gatherings, or other interactive activities, holds the power to reshape societal values and save lives simultaneously. As we continue to harness the potential of community gatherings, we elevate blood donation to more than a medical necessity [25].
Partnering with business organizations, colleges, and other institutions to establish new donors	Collaborating with business organizations, colleges, and institutions to foster new blood donors holds the promise of expanding the lifelines that sustain our communities. These partnerships infuse blood donation into the fabric of everyday life, leveraging their influence and resources to create a lasting impact. As we continue to weave these relationships, we weave together a tapestry of compassion, unity, and shared commitment towards the wellbeing of all [26].
Promotion by actors and players to engage a larger audience	The involvement of actors and players in promoting blood donation has a transformative impact that extends beyond the realm of entertainment. By leveraging their influence, these celebrities become advocates for a crucial cause, encouraging a larger audience to engage and participate [26].
Emphasis on regular donations	By fostering a reliable and consistent blood supply, this strategy guarantees the timely and dependable fulfilment of healthcare requirements. As contributors evolve into advocates of unwavering dedication, they sculpt a forthcoming era in which each unit of blood embodies a vital link, exemplifying the fortitude of human unity and empathy. Through this transformative focus on recurring contributions, we unite as guardians of existence, connected by the common goal of leaving a lasting imprint on the health and wellness of our communities [27].

Importance of blood donation and its impact on healthcare

Blood donation is an essential aspect of healthcare. It has been used in various medical procedures such as complications during childbirth, cancer treatment, burns, surgeries, accidents, and genetic blood disorders [28,29]. Several studies have designated that most of the blood, when donated by adults, is transfused to elderly patients in need [30-34]. Excessive use of blood at present could result in a shortage in the future, as numerous medical procedures such as surgery, trauma, and cancer treatments often necessitate significant quantities of blood. Therefore, everyone must recognize the importance of blood donation [27]. Donating blood and utilizing blood banking by public and non-profit organizations, along with securing low-cost supplies, could assist with blood donation during emergencies [35,36].

History of blood donation

The first successful blood transfusion between dogs occurred in the 17th century, beginning the history of blood donation. However, the use of human blood transfusions did not increase significantly until the early 1900s [37]. The ABO blood types were identified in 1901 by Austrian pathologist Karl Landsteiner, a development in the transfusion medicine sector. With the help of this discovery, it became feasible to match donors and recipients more precisely, lowering the chance of transfusion responses. The first blood bank opened in New York City in 1914 [38]. This made blood more readily available to patients who needed it by facilitating its storage and transportation. The need for blood transfusions skyrocketed during World War I. This resulted in the creation of blood donor programs and mobile blood drives. The Rhesus (Rh) blood factor was identified in the 1940s [39]. The danger of transfusion responses was further decreased because of this significant advancement in transfusion medicine [40].

Blood donation eligibility requirements

The donor should fall within the age group of 18-65 years and must weigh a minimum of 45 kilograms. Blood donation relies on assessing crucial parameters, including the donor's age, weight, temperature, blood pressure, and pulse. Furthermore, to guarantee the safety and eligibility of the donation, an evaluation of the donor's overall health, sexual orientation, and travel history is also conducted [41]. Other factors influencing blood donation practice include gender, age, religion, knowledge, attitude, level of schooling, self-perceived health status, and family education are all factors to consider [9]. Blood donation is a carefully controlled practice that guarantees the safety and effectiveness of collected blood and its components, whether used as essential components of contemporary transfusion medicine, as a therapeutic approach, or as supplementary care to other clinical therapies [42]. Antibody testing is a diagnostic approach used to detect HIV infections, and its implementation can effectively reduce the risk of disease transmission [43]. In India, the practice of transfusion-transmitted infection testing encompasses screening for five distinct diseases, namely HIV-I and HIV-II, hepatitis B virus (HBV), hepatitis C virus (HCV), Malaria, and Syphilis [30,44]. The eligibility criteria for blood donation among lesbian, gay, bisexual, and transgender (LGBTQ) individuals exhibit variation across different countries. In several nations, LGBTQ individuals may be eligible to donate blood provided they have not engaged in sexual activity with a new partner in the preceding three months [45]. Healthcare professionals should obtain the appropriate consent from the donor and recipient before blood donation [46].

**Figure 3:**
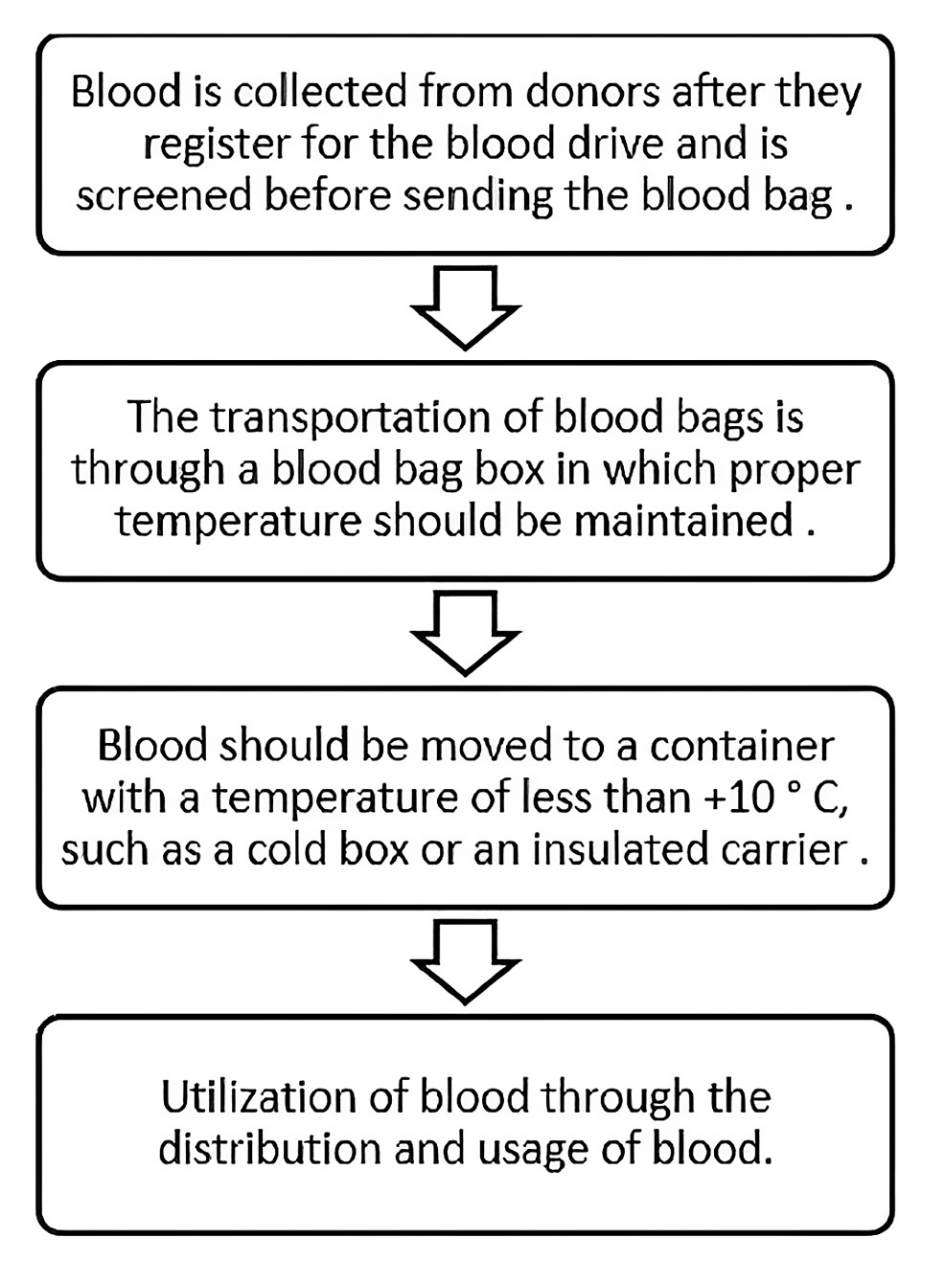
Steps involved in the blood donation process. °C: Degree Celsius. Figure composed based on [40-46].

Figure 3 shows the steps involved in the blood donation process [40-46]. Once donors register for the blood drive, their blood is carefully collected, following stringent protocols to maintain donor safety. Subsequently, the collected blood undergoes thorough screening processes before it is dispatched in sealed blood bags to ensure its suitability for transfusion [40,41]. Blood bags are transported within specialized blood bag boxes designed to uphold the crucial temperature conditions required for preserving the integrity of the blood products during transit. This temperature control ensures that the blood remains safe and effective for medical use [41,42]. To maintain blood product safety, transferring the blood to a container with a temperature of less than +10 °C, such as a cold box or an insulated carrier, is essential. This controlled temperature environment helps preserve the quality and effectiveness of the blood during transportation to medical facilities for patient care [43,44]. Blood is effectively utilized through a well-organized system of distribution and its subsequent usage in medical procedures. This critical process ensures that donated blood reaches those in need, saving lives in healthcare settings worldwide [45,46].

Awareness campaigns and initiatives

Blood donation awareness campaigns are vital for educating people about the importance of donating blood and ensuring a steady supply. These campaigns involve creating impactful visuals and leveraging social media for broad outreach [47]. Different initiatives, such as blood donation camps, can increase blood donation [48]. Repeated donors can educate young people about the need to be regular blood donors, and the mental influence they can create can increase the rate of youth blood donation [25]. One of the projects involves utilizing various social media channels to disseminate information about blood donation, highlighting the benefits it provides to both the donor and recipients [12]. Social media has emerged as the preferred source for sending and receiving information about blood donation. Utilizing social media platforms can improve blood donation practices when there is a donor shortage [12]. Organizing donation drives in convenient locations, partnering with influencers, and conducting educational workshops help dispel myths and motivate donors [48].

Donor motivation and retention

Donor motivation and retention are essential for a stable blood supply. Sex, age, and other donor information demographic considerations influence the possibility of future donations [49]. There is variation in the level of satisfaction among blood donors according to subgroups of demographics and contribution history. In addition, satisfaction positively correlates with the motivation to return for future donations. While generosity remains the primary motivating factor for all donors, optimizing future donation campaigns to cater to specific demographic subgroups might be crucial to enhancing motivation for future contributions [50]. There is a need to educate youth about the importance of becoming regular blood donors and the significant impact they can have on their communities [50]. Enhancing donor motivation and broadening the donor pool can be achieved by introducing selfie points, allowing donors to share post-donation images on social media. Moreover, leveraging Facebook's blood donation tool, which connects users with local blood centers, could enhance donor retention and overall motivation for increased donations [11]. Blood donation benefits an individual's health by decreasing oxidative stress and preventing oxidants by increasing antioxidant enzymes such as superoxide dismutase [51]. Donating blood can also help us improve our emotional and physical health [52].

Future directions and challenges

Young individuals, typically aged 18-24, have the potential to make a substantial impact in alleviating blood shortages through active encouragement to participate in blood donation efforts [21]. Research has revealed a spectrum of factors, including attitudes, social norms, perceived control, and knowledge, that influence the willingness of young people in low-income countries to engage in voluntary blood donation, with the potential to enhance blood supply and overall national health [53]. The future directions and challenges facing blood donation include the need for more diverse donors, the impact of technological advancements on blood transfusion, and the global blood shortage. It could also examine potential solutions to these challenges, such as using artificial blood or expanding blood donor networks. Undergraduate students represent a promising demographic for future blood donation initiatives, presenting an opportunity for increasing the donor pool within the country [8]. One notable psychological impediment in the context of blood donation is the phenomenon of donation-related anxiety [54]. Lack of public knowledge and education is one of the main barriers to blood donation. Numerous individuals should understand the significance of blood donation and its role in preserving lives. Future initiatives should focus on informing and teaching people about the value of blood donation [6]. One way to streamline blood donation processes is with blood donation management software designed to bridge the communication gap between donors and recipients. This application prompts prospective blood donors to register their basic information. The system then notifies donors via messages, requesting their blood donation within a 12-hour timeframe. Such a system can significantly alleviate the challenges faced by blood banks [24]. Blood donation organizations face the ongoing challenge of attracting and retaining donors. In developing nations, recruiting donors proves difficult, necessitating enhanced access to blood donation programs. Conversely, in industrialized countries, where contributors may be less inclined to donate regularly, there is a critical need to boost donor retention. Future efforts should focus on refining retention and recruitment strategies to guarantee a consistent and safe blood supply [55]. Maintaining blood donation programs requires ensuring donors' safety. Blood collection organizations must continuously assess and enhance their safety protocols to mitigate the transmission of diseases and prevent other adverse incidents. The adoption of technology can transform current blood donation procedures [56]. Mobile apps, for example, can help with donor scheduling and recruitment, while artificial intelligence can help with blood demand forecasting and inventory management [57]. Future initiatives should focus on incorporating technology into blood donation programs to increase their effectiveness and efficiency. The marketing of blood and the discrimination against specific donor groups are just two of the moral and social questions that blood donation poses. The future of blood donation depends on addressing these challenges and implementing innovative strategies to ensure a safe and viable blood supply [26,33,55].

## Conclusions

In conclusion, raising awareness of blood donation is crucial to saving lives and promoting community health. Modern strategies for raising awareness include projects like planned community events, planned social media campaigns, strategic partnerships with indigenous organizations, and employing the influence of public figures and sports persons to successfully reach and engage a broader range of the population. Nevertheless, continuous endeavors to sustain and enhance donor engagement are in progress. It delivers tailored messages to various demographic groups, offering convenient avenues for donations and tackling prevalent misunderstandings and anxieties related to blood donation.
